# Interplay between Fatty Acid Binding Protein 4, Fetuin-A, Retinol Binding Protein 4 and Thyroid Function in Metabolic Dysregulation

**DOI:** 10.3390/metabo12040300

**Published:** 2022-03-29

**Authors:** Daniela Dadej, Ewelina Szczepanek-Parulska, Marek Ruchała

**Affiliations:** Department of Endocrinology, Metabolism and Internal Medicine, Poznan University of Medical Sciences, 61-701 Poznan, Poland; ewelina@ump.edu.pl (E.S.-P.); mruchala@ump.edu.pl (M.R.)

**Keywords:** fatty acid-binding protein 4, fetuin-A, retinol binding protein 4, thyroid hormones, obesity, type 2 diabetes, insulin resistance, atherosclerosis, metabolic diseases

## Abstract

Signalling between the tissues integrating synthesis, transformation and utilization of energy substrates and their regulatory hormonal axes play a substantial role in the development of metabolic disorders. Interactions between cytokines, particularly liver derived hepatokines and adipokines, secreted from adipose tissue, constitute one of major areas of current research devoted to metabolic dysregulation. The thyroid exerts crucial influence on the maintenance of basal metabolic rate, thermogenesis, carbohydrate and lipid metabolism, while its dysfunction promotes the development of metabolic disorders. In this review, we discuss the interplay between three adipokines: fatty acid binding protein type 4, fetuin-A, retinol binding protein type 4 and thyroid hormones, that shed a new light onto mechanisms underlying atherosclerosis, cardiovascular complications, obesity, insulin resistance and diabetes accompanying thyroid dysfunction. Furthermore, we summarize clinical findings on those cytokines in the course of thyroid disorders.

## 1. Introduction

Signalling between tissues involved in energy homeostasis and thyroid plays an essential role in the regulation of metabolism. Cytokines, including adipose tissue-derived adipokines and liver-derived hepatokines, along with thyroid hormones, integrate the inter-tissue crosstalk. Thyroid hormones maintain the basal metabolic rate, participate in thermogenesis, carbohydrate and lipid metabolism, as well as the regulation of food intake. Thyroid dysfunction predisposes individuals to metabolic disorders including obesity, type 2 diabetes, hypertension and cardiovascular complications leading to premature death [1,2,3]. However, the mechanisms involved in metabolic dysregulation in thyroid dysfunction are only partially understood [4,5,6]. To date, several adipokines were defined as factors linking thyroid function and metabolism, leptin being the best studied among them [4,7]. Leptin reduces food intake and induces energy expenditure by stimulating thermogenesis and lipolysis, while resistance to its action is associated with obesity. Leptin activates the hypothalamic-pituitary-thyroid axis, increasing the expression of thyrotropin-releasing hormone (TRH) in the hypothalamus through direct and indirect mechanisms which ultimately results in the increased secretion of thyroid hormones and enhanced whole-body catabolism [8]. Recently, fatty acid binding protein type 4 (FABP4), fetuin-A and retinol binding protein type 4 (RBP4) emerged as adipokines with detrimental metabolic effects and as potential biomarkers of type 2 diabetes and cardiovascular complications [9,10,11,12,13,14,15,16,17]. Changes in their concentrations were observed in obesity, glucose intolerance, dyslipidaemia as well as thyroid diseases. This review will focus on FABP4, fetuin-A, RBP4 and their relation to thyroid function and metabolic diseases.

## 2. FABP4 and Hypothalamic-Pituitary-Thyroid Axis—Experimental Studies

Fatty acid binding protein 4, also referred to as adipocyte FABP (A-FABP) or adipocyte protein 2 (aP2), is one of the major adipocyte proteins. Apart from adipocytes that secrete the majority of circulating FABP4, it is also expressed in endothelial cells and macrophages. It acts as a lipid chaperone, involved in the intracellular transport of fatty acids and cell membrane remodelling [18]. FABP4 augments lipolysis, through interactions with hormone-sensitive lipase (HSL) and comparative gene identification-58 (CGi-58), co-activator of the adipose triglyceride lipase (ATGL) [19]. Moreover, FABP4 affects glucose metabolism in that it stimulates hepatic gluconeogenesis, inhibits glucose uptake and utilization in the peripheral tissues [20]. FABP4 aggravates insulin resistance by downregulation of peroxisome proliferator-activated receptor-γ (PPAR-γ) [21]. Current research indicates that FABP4 plays a substantial role in diabetes and cardiovascular diseases. In humans, increased circulating FABP4 concentrations were observed in metabolic syndrome, obesity, insulin resistance, type 1, type 2 and gestational diabetes, hypertension, dyslipidaemia, myocardial dysfunction and renal failure [22,23,24,25].

FABP4 is involved in the development of atherosclerosis: it stimulates foam cell formation, inducing free fatty acids’ phagocytosis in macrophages, and exerts a pro-inflammatory response, promoting endothelial dysfunction [20]. FABP4 expression associates with plaque instability [26]. Its serum concentrations correlate with the risk of adverse cardiovascular events, including myocardial infarction and stroke, and are indicative of poor outcomes following ischemic stroke [19,27]. Importantly, FABP4 concentration was proved to be a predictor of CVD-related morbidity and mortality [14,19].

Carotid intima media thickness (IMT) is widely used as a simple, reproducible and non-invasive surrogate for atherosclerosis and subclinical cardiovascular disease [28]. A single study by Tan et al. assessed the correlation between FABP4 and IMT in hypothyroid subjects [29]. They demonstrated that IMT was positively associated with TSH and significantly higher in hypothyroid patients compared to healthy subjects. FABP4 was also higher in hypothyroid patients and correlated with TSH. Nonetheless, no correlation between FABP4 and IMT was found. Research on IMT in thyroid dysfunction, though extensive, yielded contradictory results [30]. Current data concerning FABP4 and IMT are as well limited and inconclusive; several studies support a positive association between FABP4 and IMT [31,32,33], while others failed to identify such correlations [34,35]. Further randomized controlled trials are needed to clarify the relation between IMT, FABP4 and thyroid function.

In prospective studies, FABP4 was identified as an independent predictor of type 2 diabetes and insulin resistance [13,19,36]. A recent study by Prentice et al. indicates that FABP4 may be involved in the pathogenesis of diabetes [24]. The study identified a new hormone—“fabkin”, composed of FABP4 and two extracellular nucleoside kinases: adenosine kinase (ADK) and nucleoside diphosphate kinase (NDPK), that regulates the function of pancreatic β-cells. Briefly, FABP4 binding alters the activity of both kinases, which determines the availability of extracellular adenosine triphosphate (ATP) and adenosine diphosphate (ADP)—an agonist of purinergic receptor P2Y1 localised on the β-cell surface. In the presence of FABP4, ADK is activated and NDPK inhibited, resulting in low ADP/ATP ratio, which inhibits surface receptors and consequently decreases glucose-stimulated insulin secretion. Acting through P2Y1 receptors, “fabkin” modulates cyclic adenosine monophosphate (cAMP), which disrupts endoplasmic reticulum calcium homeostasis, exposing β-cells to environmental stress and preterm death. Targeting “fabkin” with antibodies had a protective effect on β-cell function and mass, and prevented the development of diabetes.

Thyroid hormones participate in pancreatic β-cell differentiation and regulate their function [37]. Studies in animal models demonstrated that triiodothyronine (T3) administered in the early postnatal period increased β-cell proliferation and maturation, which is mediated by the interaction of thyroid receptors with the MAF bZIP transcription factor A (MAFA) gene promoter and results in improved glucose dependent insulin secretion [38]. T3 was shown to preserve β-cell viability and to augment insulin secretion in cell cultures through binding to thyroid receptor β1, which further activates the phosphatidylinositol 3 kinase/protein kinase B pathway [39,40]. Additionally, studies in animal models indicate that thyroid hormone deficiency may induce oxidative stress that results in degenerative changes to β-cells and their dysfunction [41,42]. The current research lacks information as to whether FABP4 and thyroid hormones interact in the regulation of pancreatic β-cell function.

The results of a recent study by Nie et al. in euthyroid subjects suggest that FABP4 may affect the sensitivity to thyroid hormones [43]. FABP4 concentrations were associated with indices used to assess central and peripheral sensitivity to thyroid hormones: thyroid feedback quantile-based index (TFQI), thyroid stimulating hormone index (TSHI) and T3/T4 ratio, in that increased FABP4 concentrations were indicative of a decreased sensitivity. Recent findings indicate that reduced sensitivity to thyroid hormones may correlate with diabetes, hypertension, metabolic syndrome and non-alcoholic fatty liver disease (NAFLD) [44,45,46,47,48]. The precise mechanisms by which FABP4 could affect the sensitivity to thyroid hormones are largely unknown.

On the other hand, in brown adipose tissue, (BAT) FABP4 was identified as an essential factor that warrants thyroxin (T4) to T3 conversion and thus thyroid hormone activity in target cells, such as adaptive thermogenesis stimulation [49]. T4 is a predominant circulating thyroid hormone which requires conversion into biologically active T3. This process occurs in peripheral tissues, catalysed by type 1 (DIO1) or type 2 5′deiodinases (DIO2) and is regulated by substrate availability (reflecting thyroid function), thyroid receptors and enzyme activity [50]. Under thermogenic stimuli, the DIO2 expression in BAT increases and results in enhanced conversion of T4 to T3, which in turn promotes thermogenesis [51]. Shu et al. found that FABP4 knock-out mice exposed to cold or high fat diets demonstrated lower DIO2 expression, lower T3 and reduced thermogenesis, and energy expenditure compared with wild-type mice [49]. This effect was attenuated by the administration of exogenous FABP4, but not T4. FABP4 promoted the expression of DIO2 by suppressing liver X receptor α (LXR). LXR are the nuclear receptors, functioning as ligand-regulated transcription factors, which are key to lipid and glucose homeostasis [52,53,54]. LXR influence hypothalamic-pituitary-thyroid axis through the inhibition of thyrotropin releasing hormone in the paraventricular nucleus of hypothalamus as well as by reducing the expression and activity of DIO2 in adipose tissue [55,56]. Whether FABP4 could regulate thyroid hormone activity by targeting LXR outside BAT requires further exploration in future studies.

Preclinical studies demonstrated the potential utility of targeting FABP4 in the treatment of metabolic disorders. A variety of small molecule inhibitors as well as antibodies directed against FABP4 were developed. The pharmacological inhibition of FABP4 in mice improved glucose tolerance [57,58], attenuated adipose tissue, skeletal muscle and macrophage inflammation [59,60], and alleviated endothelial dysfunction and atherosclerosis [61] and NAFLD [62]. In human studies, widely used angiotensin II receptor blockers [63], atorvastatin [64], omega-3 fatty acids [65] and sitagliptin [66] were found to decrease FABP4 concentrations.

## 3. Fetuin-A and Hypothalamic-Pituitary-Thyroid Axis—Experimental Studies

Fetuin-A, also known as α-2 Heremans-Schmid glycoprotein (AHSG), is synthesised and secreted mainly from the liver and to a lesser extent from adipose tissue. Therefore, it can be termed both as hepatokine and adipokine [67]. It belongs to the superfamily of cystatin proteins and encompasses two polypeptide chains—the heavy A chain and light B chain, connected by an interchain disulfide bridge which results in a loop structure [68]. The protein structure allows molecule binding, transport, and receptor interactions. Fetuin-A exerts multiple physiologic functions including the regulation of calcium homeostasis, bone mineralization and osteogenesis, fatty acid and protein metabolism, and regulation of inflammatory responses. Due to its deleterious effects on insulin sensitivity and glucose metabolism, it has raised interest as a molecular link between insulin resistance, obesity, NAFLD and metabolic syndrome. Serum fetuin-A concentrations are relatively high and influenced by dietary factors [69,70], physical activity [71] and body composition [67]. Increased concentrations were observed in obesity [72], metabolic syndrome [73,74], type 2 diabetes [75], NAFLD [76] and may serve as a predictor of type 2 diabetes [16,17,77]. Serum fetuin-A concentration may be also considered as a cardiovascular risk factor [78,79,80].

Fetuin-A regulates calcium homeostasis. It has the ability to bind the surplus of calcium and phosphate to form calciprotein particles (CPP). The CPP then form larger aggregates, amorphous calcium phosphate-rich nanoparticles, composed of calcium, phosphate, fetuin-A and matrix Gla protein (MGP) which can be further eliminated [81]. Thereby, fetuin-A increases calcium clearance and inhibits extraosseous calcification [82,83]. Calcification of coronary arteries (CAC) increases the cardiovascular risk and its progression associates with cardiovascular events both in observational and prospective studies [84,85]. Dystrophic deposition of calcium in atherosclerotic plaques exacerbates atherosclerosis [86,87]. Calcium accumulates in vascular smooth muscle cells (VSMC) and pericytes, which promotes apoptosis and local inflammation resulting in plaque progression and rupture due to impaired stability [88,89]. Fetuin-A was demonstrated to reduce the calcium deposition in VSMC, preventing their calcification and apoptosis [90]. Serum fetuin-A levels are inversely associated with the severity of arterial calcification [91,92,93], the presence of coronary artery disease (CAD) [94,95,96], cardiovascular mortality [80,97,98], as well as all-cause mortality in patients with CAD [80,99]. On the contrary, several studies demonstrated that fetuin-A promotes CAD and have shown a positive correlation between fetuin-A and the incidence and severity of CAD [79,100,101,102]. These contradictory results emphasise the manifold effects of fetuin-A on the cardiovascular system. On one hand low fetuin-A concentrations result in vascular calcification and the progression of atherosclerosis, and CAD, while on the other hand high fetuin-A promotes insulin resistance, local inflammation and progression of lipid pools, which may also exacerbate atherosclerosis.

Thyroid hormones act together with fetuin-A to prevent soft tissue calcification. T3, in physiological concentrations, was demonstrated to increase the expression of MGP—a potent inhibitor of calcification in VSMC through thyroid hormone nuclear receptors, which prevents vascular calcification [103]. Low serum T3 was found to correlate inversely with CAC in euthyroid subjects [104,105], while low T4 was related to high risk of CAC progression [106]. Several studies indicated that low TSH was associated with a greater degree of CAC in euthyrodism and subclinical hyperthyroidism [107,108]. Despite an incomplete understanding of the mechanisms at the cellular and cytokine levels, the detrimental effects of thyroid dysfunction on cardiovascular risk, including CAD, are well established [109].

Fetuin-A contributes to the development of insulin resistance. It inhibits the insulin induced autophosphorylation of tyrosine kinase of the insulin receptor, which suppresses insulin signalling in the liver, muscle and adipose tissue. This leads to a decreased response to insulin in insulin-sensitive tissues and triggers insulin resistance [110,111,112]. Additionally, fetuin-A promotes lipid-induced insulin resistance. It enhances free fatty acids (FFA) binding to toll-like receptor 4 (TLR-4) in adipocytes, which activates nuclear factor κ-B (NF-κB) and activator protein 1 that further upregulate the expression of inflammatory genes, leading to the increased synthesis of proinflammatory cytokines, adipose tissue inflammation and consequently to insulin resistance [113]. FFA induced overexpression of fetuin-A leads to recruitment and migration of macrophages to adipose tissue and their polarization towards the M1 pro-inflammatory secreting subtype, which exacerbates inflammatory reactions [114]. Fetuin-A was also found to downregulate the adiponectin expression in adipocytes through the Wnt3a/PPAR pathway, which antagonizes the insulin-sensitizing and anti-inflammatory properties of adiponectin [115,116]. Fetuin-A was also demonstrated to impair pancreatic β-cell function directly by mediating lipotoxicity. In lipid excess, FFA induce the secretion of fetuin-A from β-cells. Both circulating and β-cell-derived fetuin-A aggravate inflammation, disrupt insulin secretion and promote β-cell apoptosis [117,118,119]. Consistent with those findings, fetuin-A knock-out mice demonstrated augmented insulin sensitivity and were resistant to diet-induced weight gain [120]. The association between increased serum fetuin-A concentration, insulin resistance and type 2 diabetes was also confirmed in human trials [121,122]. Several studies demonstrated a potential utility of fetuin-A to predict the risk of type 2 diabetes [15,16,17,77].

As previously stated, thyroid hormones regulate β-cell function and influence insulin sensitivity, while thyroid dysfunction, both overt and subclinical, predisposes to obesity, insulin resistance and type 2 diabetes [123,124]. Current research indicates that lipotoxicity may in turn affect thyroid function, causing deleterious changes to the thyroid gland and subsequently resulting in hypothyroidism [8]. Studies in obese and diabetic Wistar rats demonstrated a reduction in inflammatory cytokine expression and inflammatory cell infiltration in adipose tissue as well as an improvement in insulin sensitivity upon treatment with T3 [125,126]. Other studies implied that T3 administration to diabetic mice improves insulin sensitivity in an inflammatory-independent manner [127]. Thyroid hormones are also likely involved in the regulation of adiponectin expression in adipose tissue [128,129,130,131]. Both animal and human studies suggest that adiponectin concentration changes in thyroid dysfunction, however results are inconclusive [129,132,133,134,135,136,137]. Taking the above into consideration, thyroid hormones and fetuin-A share common pathways in the regulation of insulin sensitivity.

Recent evidence suggests a direct influence of thyroid hormones on fetuin-A expression. Thyroid status alters protein synthesis in the liver by modulation of RNA polymerase activity. Hyperthyroidism was found to be associated with increased protein synthesis, while hypothyroidism with decreased [138]. An in vitro study revealed the stimulatory effect of T3 on fetuin-A expression in the liver cell line. The molecular mechanism involves T3 binding to thyroid receptor α1, which then regulates the transcription of various proteins including fetuin-A, by affecting gene promoter regions [139]. Consistent with this assumption, T3 administration to hypophysectomized rats increased serum fetuin-A levels [140]. A human study in patients undergoing stimulation with recombinant human TSH (rhTSH) as screening for recurrence of thyroid cancer demonstrated no changes in serum fetuin-A after rhTSH administration [141]. In the majority of clinical studies, as discussed in further paragraphs, fetuin-A concentration correlates with TSH. We hypothesize that TSH may not directly influence fetuin-A concentration, but rather reflects a long-lasting hypothyroid or hyperthyroid state, which in other mechanisms, likely involving T3, alter fetuin-A concentration.

## 4. RBP4 and Hypothalamic-Pituitary-Thyroid Axis—Experimental Studies

Retinol Binding Protein 4 (RBP4) is a protein from the lipocalin family that is primarily involved in the transport and regulation of vitamin A. Lipocalins have a unique structure—‘lipocalin fold’, that determines the binding of hydrophobic ligands [142]. RBP4 is synthesised predominantly in the liver, but other tissues including adipose tissue also secrete RBP4 [143]. Therefore, like FABP4 and fetuin-A, it might be also referred to as adipokine. In the circulation, RBP4 forms compounds with transthyretin, which prevents RBP4 catabolism and filtration in the kidney [144]. Nutritional status may affect the production of RBP4 [145]. Increased RBP4 concentrations were found in diabetes mellitus [146,147], dyslipidaemia [148], cardiovascular diseases and obesity, particularly visceral adiposity [143], and were associated with the increased risk of metabolic syndrome development [74]. RBP4 correlated significantly with lipid profile in that its increased concentration was associated with high triglyceride and low HDL levels, which are major compounds of atherogenic dyslipidaemia [149,150,151]. RBP4 has been demonstrated to induce oxidative stress and inflammation, leading to endothelial dysfunction and foam cell formation, thereby promoting atherosclerosis [152,153,154]. Most of the current research corroborates the usefulness of RBP4 as a biomarker of cardiovascular diseases. Increased RBP4 concentrations correlate with established cardiovascular risk factors [10] as well as ischemic cardiovascular incidents [9,155], and could serve as a marker of cardiovascular disease in both diabetic [156], and non-diabetic subjects [157]. However, opposing results were also reported [158,159,160].

Thyroid function is closely related to cardiovascular health. Hypothyroidism, both overt and subclinical, predisposes to the development and progression of cardiovascular diseases [161]. Dyslipidaemia and hypertension, accompanying hypothyroidism, facilitate atherosclerosis and endothelial dysfunction, which all contribute to CAD [162]. Low T3, even within the normal range, associates with the occurrence and severity of coronary atherosclerosis, and may serve as a predictor of advanced CAD [163,164]. In hypothyroid subjects, the concentration of RBP4 correlated with the incidence and severity of CAD, which was classified by the number of affected vessels and using the Gensini score. It was also positively associated with total and low density lipoprotein cholesterol, which are CAD risk factors [165]. Higher levels of RBP4 were also observed in hypothyroid patients with a positive family history of CAD compared to those without a family burden, while a positive family history was associated with cardiovascular risk factors including obesity, hypertension, hypercholesterolemia and insulin resistance [166]. Further studies are required to clarify if increased serum RBP4 has a causal contribution to the development of CAD associated with thyroid dysfunction.

RBP4 participates in the development of insulin resistance. Animal studies demonstrated elevation in serum concentrations of RBP4 in multiple mouse models of insulin resistance. RBP4-knock-out mice were less likely to develop insulin resistance, while the overexpression or exogenous administration of human RBP4 impaired glucose-stimulated insulin secretion (GSIS) and resulted in insulin resistance and glucose intolerance [147,167]. Several mechanisms have been proposed to underlie the deleterious effect of RBP4 on insulin sensitivity. Initially, RBP4 was indicated to enhance the expression of phosphoenolpyruvate carboxykinase—gluconeogenic enzyme in liver and to disrupt insulin signalling in muscle by modulating the activity of phosphoinositide 3-kinase and the availability of tyrosine-phosphorylated insulin receptor substrate-1 [147]. Then, similarly to fetuin-A, RBP4 was shown to induce adipose tissue inflammation by activating the toll-like receptors 2 and 4 (TLR-2, TLR-4) on the inflammatory cells’ surface, which further stimulates downstream pathways involving NFκB, c-Jun N-terminal kinases (JNK) and the secretion of proinflammatory cytokines, which disrupts insulin signalling in adipocytes and ultimately results in insulin resistance [168,169,170]. RBP4 was also associated with the downregulation of PPAR-γ—a negative regulator of inflammation [168]. Another hypothesis suggests that RBP4 impairs insulin signalling through the interaction with retinoic acid 6 (STRA6) receptors, known as specific receptors for RBP4, that participate in retinol transmembrane transport. In white adipose tissue and muscle, retinol-bound RBP4 binding with STRA6 induced the phosphorylation of Janus kinase 2 (JAK2) and increased the expression of signal transducer and activator of the transcription (STAT) target gene Socs3 that potently inhibits insulin signalling. In STRA6-null mice, RBP4 administration did not alter the kinase phosphorylation status or gene expression [171,172]. In pancreatic β-cells, RBP4 interaction with STRA6 resulted in the suppression of GSIS by downregulation of insulin transcription via the JAK2/STAT1/insulin gene enhancer protein (ISL-1) pathway [167]. Increased RBP4 concentrations were demonstrated to negatively correlate with pancreatic β-cell function both in animal [167] and human studies [173,174,175]. However, contrasting evidence exists [176,177]. An association between RBP4 and increased risk of type 2 diabetes was reported in healthy women [12] and subjects with prediabetes [11,178]. Its concentration also correlated with the incidence of gestational diabetes [179].

Thyroid hormones counteract RBP4 and prevent insulin resistance through reducing adipose tissue inflammation [125,126] and in inflammatory-independent mechanisms [127]. They also improve β-cell viability and enhance glucose-stimulated insulin secretion [37,38]. Thyroid hormone deficiency increases the risk of insulin resistance and diabetes. In hypothyroid subjects, RBP4 correlated positively with fasting glucose, insulin and HOMA-IR [166], which suggests that it may be involved in the pathogenesis of glucose intolerance accompanying hypothyroidism, but underlying mechanisms remain to be clarified. Several studies focused on the potential significance of transthyretin (TTR)—a protein that regulates RBP4 metabolism and simultaneously transports thyroid hormones. Increased TTR concentration was observed in glucose intolerance, contributing to high circulating RBP4 levels [180,181,182,183]. TTR plays a role in the transport of thyroid hormones, accounting for 10% of bound T4. However, the majority of thyroid hormones are bound to another carrier protein, thyroxine-binding globulin (TBG). Moreover, variations in binding proteins affect total concentrations of thyroid hormones, but not the amount of unbound, metabolically active hormones [184]. Therefore, TTR is not associated with thyroid function and could not link it to RBP4.

The majority of recent studies confirm that higher RBP4 concentrations are associated with impaired glucose metabolism and hyperinsulinemia, and therefore RBP4 was investigated as a potential therapeutic target. Several direct RBP4 antagonists were found to decrease RBP4 serum concentrations [185]. Currently used anti-diabetic drugs, including rosiglitazone [147] and sitagliptin [186,187], were also found to reduce RBP4 concentrations. The clinical significance of those findings remains to be evaluated.

**Figure 1:**
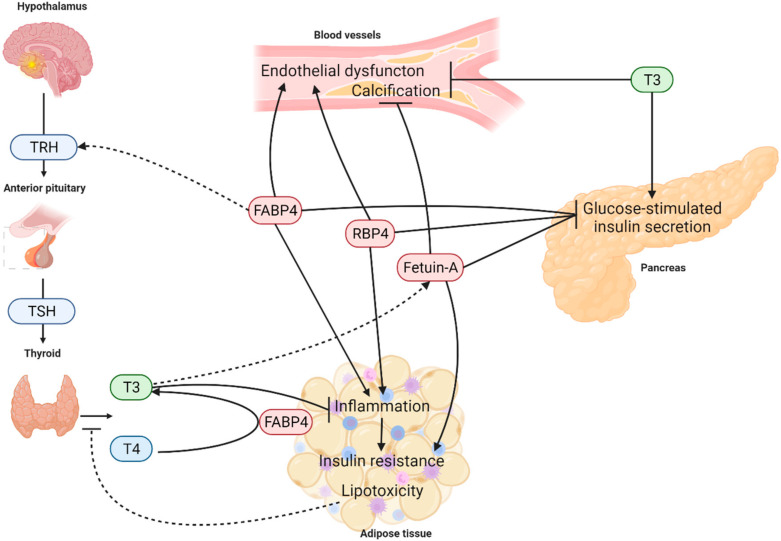
Interactions between FABP4, fetuin-A, RBP4 and hypothalamic-pituitary-thyroid axis. FABP4—fatty acid binding protein 4, RBP4—retinol binding protein 4, TRH—thyrotropin releasing hormone, TSH—thyroid stimulating hormone, T3—triiodothyronine, T4—thyroxine. Known interactions are presented as solid lines, while possible interactions are indicated with dotted lines. Arrows indicate a stimulating effect or process. Lines ending with vertical lines stand for inhibition.

The interactions between analysed adipokines and thyroid hormones are shown in Figure 1.

## 5. Adipokines and Hypothalamic-Pituitary-Thyroid Axis—Clinical Studies

Alterations in circulating FABP4 were demonstrated in thyroid dysfunction. Increased FABP4 concentrations were observed in patients with overt hyperthyroidism compared to euthyroid subjects, which then decreased along with the restoration of normal thyroid function [188,189]. Similarly, individuals with overt and subclinical hypothyroidism presented higher serum FABP4 concentrations compared with healthy controls [29]. In those studies, FABP4 concentration correlated positively either with T3 [188], T4 [188,189] or TSH [29]. In euthyroid subjects with Hashimoto thyroiditis, FABP4 concentrations were higher than in healthy controls and were directly associated with T4 and insulin concentration [190]. The authors identified no association between FABP4 and thyroid hormones or insulin in the control group without Hashimoto thyroiditis, suggesting that the increase in FABP4 is attributable to autoimmunity itself, as hormonal status did not differ between Hashimoto sufferers and healthy subjects. On the other hand, in patients with Graves’ orbitopathy, FABP4 concentration did not differ compared with healthy controls [191].

Several studies assessed fetuin-A serum concentrations in thyroid diseases. In hyperthyroid subjects, serum fetuin-A was significantly higher compared to those in the euthyroid state [188,192,193], and correlated negatively with TSH [193] or log transformation of TSH (logTSH) [192]. On the contrary, in subjects with overt hypothyroidism, fetuin-A concentrations were lower than in healthy controls and also associated inversely with TSH [194]. Results concerning subclinical hypothyroidism are inconclusive [195,196]. The restoration of normal thyroid function diminished the differences between patients with thyroid dysfunction and euthyroid study groups [192,193,194]. A cross sectional study in a large Chinese cohort supports the association between serum fetuin-A and thyroid function, in that subjects in the highest free (f)T3 and fT4 terciles had higher fetuin-A levels compared to ones in the lowest fT3 and fT4 terciles. What is more, the study demonstrated a positive association between log transformation of fT3 and fetuin-A concentration [197].

Alterations in circulating RBP4 levels accompanying thyroid disorders were demonstrated. Increased RBP4 concentrations were observed in patients with both subclinical and overt hypothyroidism compared to euthyroid individuals [165,166,198,199]. In hypothyroid individuals, RBP4 correlated positively with TSH [165,166,198,199], and negatively with T3 [165], which indicates a potentiation of the stimulating effect of hypothyroidism on RBP4 with its increase in severity. Similarly, the achievement of euthyroidism with treatment led to a substantial decrease in RBP4 [199]. Increased RBP4 concentrations were also reported in euthyroid Hashimoto’s subjects. However, in this group, RBP4 was positively related to free thyroid hormones [190]. In hypothyroidism, RBP4 correlated positively with the lipid profile, fasting glucose, insulin and HOMA-IR [166], while in Hashimoto thyroiditis it correlated with insulin and HOMA-IR [190]. Studies in hyperthyroidism yielded contradictory results. Kokkinos et al. have shown a tendency towards higher RBP4 concentrations in hyperthyroid subjects compared to euthyroid controls, yet are without statistical significance. However, restoration of the euthyroid state led to a significant decrease in RBP4 [199]. Steinhoff et al. demonstrated lower RBP4 concentrations in hyperthyroid states compared to the restoration of euthyroidism within the same group. The study lacked in the euthyroid control group and was carried out in a small group of 19 patients [188]. In subjects with Graves’ orbitopathy, RBP4 concentration was not associated with clinical activity score (CAS), but correlated positively with total eye score assessed according to modified NOSPECS classification [200]. The study involved both euthyroid and hyperthyroid patients with Graves’ disease, but offered no comparison of adipokine concentration between those groups. Nonetheless, the study failed to identify any relationship between RBP4 and hypothalamic-pituitary-thyroid axis hormones.

Changes in adipokines’ concentrations observed in thyroid disorders are summarised in Table 1. Overall, the results indicate that thyroid status and circulating FABP4, fetuin-A and RBP4 are related, thus suggesting that these adipokines may be involved in the development of metabolic complications accompanying thyroid dysfunction. Animal studies have shown that adipokines’ downregulation has a beneficial effect on carbohydrate and lipid metabolism, while reduction in their concentration, achieved with antidiabetic and antihypertensive drugs, was observed in humans. Therefore, therapies targeting FABP4, fetuin-A and RBP4 may prove to be effective means of counteracting metabolic complications in thyroid disorders.

## 6. Conclusions

Current research indicates that FABP4, fetuin-A and RBP4 interact with thyroid hormones in the regulation of the body’s metabolism and may be involved in the metabolic dysregulation associated with thyroid dysfunction. The analyzed adipokines contribute to the development of insulin resistance, atherosclerosis and pancreatic β-cell dysfunction, counteracting the beneficial effects of thyroid hormones. Moreover, adipokines and hypothalamic-pituitary-thyroid hormones mutually regulate their synthesis and activity. Clinical studies support these hypotheses, in that circulating adipokines’ concentrations are affected by thyroid status and are associated with the hypothalamic-pituitary-thyroid axis. Defining the role of FABP4, fetuin-A and RBP4 presents a promising direction for the prevention and treatment of metabolic consequences accompanying thyroid disorders.

## Figures and Tables

**Table 1:** Summary of studies assessing FABP4, fetuin-A and RBP4 concentrations in thyroid disorders.

Adipokine	Thyroid Disorder	Adipokine Concentration	*p* Value ^1^	Correlated HPT-axis Parameters	Correlation Coefficient	*p* Value	Reference
FABP4	Hypothyroidism	↑	0.0140	TSH	0.201 ^b^	0.039	[29]
	Hyperthyroidism	↑	0.016 *	fT4 fT3	0.27 ^d^ 0.28 ^d^	0.037 0.031	[188]
		↑	0.038	fT4	2.51 ^c^	0.018	[189]
	Hashimoto thyroiditis	↑	<0.001	fT4	0.131 ^b^	<0.001	[190]
	Graves orbitopathy	no change		NA			[191]
Fetuin-A	Hypothyroidism	↓	0.0001	TSH	−0.61 ^a^	0.001	[194]
		↑	0.019	NA			[195]
		↓	0.001	NI			[196]
	Hyperthyroidism	↑	<0.0001 *	TSH fT4	−0.553 ^b^ 0.473 ^b^	0.0001 0.002	[193]
		↑	0.018 *	NI			[188]
		↑	0.010	logTSH	53.79^c^	0.010	[192]
RBP4	Hypothyroidism	↑	<0.0001	TSH	0.241 ^a^	0.001	[198]
		↑	<0.001	TSH	0.5 ^a^	<0.001	[199]
		↑	0.03	TSH	0.389 ^b^	0.03	[166]
		↑	<0.001	TSH T3	0.257 ^b^ −0.247 ^b^	<0.001 <0.001	[165]
	Hyperthyroidism	no change					[199]
		↓	0.048 *	NI			[188]
	Hashimoto thyroiditis	↑	<0.001	fT3 fT4	0.077 ^b^ 0.093 ^b^	0.007 0.003	[190]
	Graves’ orbitopathy	no difference GO vs. no GO		NI			[200]

^1^ compared to healthy controls/* restored euthyroid state within the same group; HPT—hypothalamic-pituitary-thyroid; FABP4—fatty acid binding protein 4; RBP4—retinol binding protein 4; ↑—increase; ↓—decrease; GO-Graves orbitopathy, ^a^—r Pearson, ^b^—r Spearman, ^c^—β regression coefficient, ^d^—Kendall τ coefficient, NA—not available, NI—not identified.

## Data Availability

Not applicable.
